# The mediating role of FoMO and the moderating role of narcissism in the impact of social exclusion on compulsive buying: a cross-cultural study

**DOI:** 10.1186/s41155-023-00274-y

**Published:** 2023-11-07

**Authors:** Merve Mert, Dilaver Tengilimoğlu

**Affiliations:** 1 Istanbul Nisantasi University, Istanbul, Turkey; 2 Atılım University, Ankara, Turkey

**Keywords:** Social exclusion, Compulsive buying, Fear of missing out (FoMO), Narcissism, Cross-cultural

## Abstract

**Background:**

There is an interrelationship between the concepts of social exclusion, compulsive buying behavior, fear of missing out (FoMO), and narcissism. Nevertheless, the extent to which these concepts mediate or moderate their relationships with each other has not been efficiently investigated.

**Objective:**

This research aims to investigate how FoMO mediates and narcissism moderates the correlation between social exclusion and compulsive buying behavior. In addition, the research aims to test a conceptual model and highlight the differences that may occur in the conceptual model proposed in two different countries.

**Methods:**

This model was analyzed among 1007 university students (Turkey = 506, Denmark = 501). The study used scales to measure social exclusion, compulsive buying behavior, FoMO, and narcissism. The study employed PROCESS Model 4 to analyze direct and indirect (mediation) effects and PROCESS Model 59 to assess conditional (moderation) effects. Furthermore, the Johnson-Neyman technique was utilized to investigate interaction terms.

**Results:**

The findings indicate that those who face social exclusion tend to participate more in compulsive buying, and this connection is partly explained by FoMO. This suggests that individuals who encounter social exclusion may have an increased likelihood of experiencing FoMO, which may subsequently contribute to compulsive buying behavior. Furthermore, the moderating effect of narcissism differed between the Turkey and Danish samples. Specifically, in the Turkey sample, narcissism only modified the connection between social exclusion and FoMO, while in the Danish sample, it impacted both the connection between social exclusion and FoMO and the connection between FoMO and compulsive buying.

**Conclusion:**

The obtained results show that the regulating role of narcissism is different in Turkey and Denmark within the conceptual model we studied.

## Introduction

Social media is currently the most commonly used Internet platform and is considered one of the most significant means of interaction, enabling rapid communication and connection between individuals (Kircaburun, 2016). We Are Social Digital’s 2022 report shows 4.62 billion social media users globally, representing 58% of the world’s population (We Are Social & Hootsuite, 2022). According to the report, the annual growth rate of social media users was found to be 13.2%. The main reasons for this growth are that it allows users to follow breaking news and developments, access entertaining content, spend their free time, keep in touch with friends, and track what they are doing (We Are Social & Hootsuite, 2022). Although social media is coming to the forefront with its positive attributes, it also has negative effects on individuals. Previous research shows that social media can lead to academic failure, distraction, sleep problems, and physical health problems (Alt, 2015; Boustead & Flack, 2021; Kartol & Gündoğan, 2020). In addition, some research shows that social media can cause various negative emotions such as jealousy, loneliness, depression, low life satisfaction, feelings of inadequacy, and fear of social exclusion (Acar et al., 2020; Deniz, 2021; Elhai et al., 2020; Servidio, 2021). Furthermore, some studies have found that these negative emotional states can also affect an individual’s buying behavior (Aydin et al., 2021; Çelik et al., 2019). Kukar-Kinney et al. (2016) found that negative emotions triggered by social media are associated with compulsive buying behavior. Similarly, Mead et al. (2011) found that people who feel socially excluded, which is a negative emotional state, may make compulsive purchasing decisions.

According to Krych’s (1989) definition, compulsive buying behavior is buying behavior that turns buying into an obsession. Compulsive buyers show a repeated and overwhelming urge to buy goods that sometimes do not work. This should be explained by considering the psychological and emotional dimensions of compulsive buying behavior (Harnish & Bridges, 2015). Individuals may resort to compulsive buying behaviors to cope with unwanted negative moods and to relieve negative emotions (Faber & Christenson, 1996). In other words, a negative affect can serve as a trigger that can mobilize an individual’s compulsive buying tendency (Yi, 2012).

The experience of social exclusion can be a driving force that causes people to engage in compulsive buying behavior. Social exclusion is defined as invisibility and separation from the social interactions of others (Knowles & Gardner, 2008). According to Williams and Jarvis (2006), social exclusion not only increases the risk of mental disorders and negative emotional states but also threatens individuals’ fundamental needs such as self-esteem, sense of belonging, purposeful existence, and control. Previous research has shown that such threats motivate people to a significant degree of negative behavior (Alabri, 2022; Han, 2020). Again, some studies have shown that consumers who experience social exclusion tend to engage in compulsive buying behavior to get rid of their negative feelings and feel good about themselves (Aydin et al., 2021; Çelik et al., 2019). For example, an individual who feels socially excluded looks for ways to relieve himself and turns to compulsive buying behavior that gives him relief from his negative feelings (Good & Hyman, 2020; Han, 2020). On the other hand, to escape the fear of social exclusion, individuals can resort to the strategy of closely observing their environment, as proposed in Noelle-Neumann’s (1977) theory of the silence spiral, often aiming development of FoMO (Holte et al., 2022). It is therefore to be expected that an individual who is constantly under social scrutiny will resort to compulsive buying behavior in order to get rid of these negative feelings. The severity of these orientations may be influenced by the personality traits of the individual. Narcissism, defined as a personality trait that reflects depersonalization toward others, may influence these tendencies (Hung, 2014). Narcissistic individuals may exhibit less compulsive buying behavior in correcting their negative emotional states because they tend to ignore their negative emotional states and perceive themselves as superior. Thus, the strength of the direct and indirect association between social ostracism and compulsive buying behavior may be moderated by narcissistic personality traits. However, the relationship between social ostracism and compulsive buying behavior and the potential mediating and regulating factors that influence this relationship have not been thoroughly investigated. Investigating these mediating and regulating mechanisms is critical to better understanding compulsive buying behavior and to developing effective strategies for intervention and prevention. The aim of this study is to investigate the mediating role of FoMO in the relationship between social exclusion and compulsive buying behavior and the moderating role of narcissism in this context. In addition, the study aims to test a conceptual model and highlight the differences that might emerge in the proposed conceptual model in Turkish and Danish samples.

### Social exclusion and compulsive cuying

The experience of feeling socially excluded is quite common among people (Knowles & Gardner, 2008; Williams and Jarvis, 2006). In fact, every individual experiences social exclusion to some degree at least once a day (Alabri, 2022). This feeling causes psychological problems such as social anxiety, depression, and hopelessness in the individual, and the individual exhibits uncontrolled behaviors to cope with these negative emotions (Alabri, 2022; Lim, 2019). In other words, social exclusion may cause the individual to unconsciously behave like others or impulsively buy the products they have (Lakin et al., 2008). The literature identifies two main reasons for this situation. According to Leary (1990), the first reason is that individuals who are left alone suggest shopping as a buffer for the frustrations of exclusion. According to Mead et al. (2011), the second reason is that individuals experiencing social exclusion impulsively store to feel like they belong to a group. In summary, Leary (1990) and Mead et al. (2011) show that people experiencing social exclusion might make impulsive purchasing decisions. The theoretical research suggests that a similar mechanism is at play in the connection between social exclusion and the tendency to compulsive buying behavior. Similar to the connection between social exclusion and impulsive buying, research has demonstrated that consumers who experience social exclusion exhibit a significant level of negativity (Alabri, 2022). In addition, it is known that cognitive distortions increase when they feel the need to belong (Şimşek et al., 2021). This situation can lead to compulsive buying behavior in individuals, which is associated with uncontrollable motives. In other words, fear of social exclusion may be a driving force for compulsive buying behavior, and the propensity to engage in compulsive buying behavior may depend on whether social exclusion is experienced (Han, 2020; Harnish & Bridges, 2015).

### The mediating role of FoMO

In essence, FoMO refers to a psychological phenomenon characterized by feelings of intense anxiety that individuals may experience when they perceive that they are missing out on enjoyable experiences in situations or environments where they are not present (Przybylski et al., 2013). It has been demonstrated by numerous studies that FoMO can emerge as a consequence of negative experiences (Duman & Ozkara, 2021; Elhai et al., 2018; Holte & Ferraro, 2020; Holte et al., 2022; Liu and Ma, 2020; Salem, 2015). Zhang et al. (2021) discovered that individuals who have a fear of social exclusion often encounter negative situations that lead to the experience of FoMO, resulting in them being in a state of constant social monitoring. Moreover, it is known that people tend to imitate others’ behaviors, attitudes, and consumption styles to eliminate the negative feelings they experience (Alt, 2015). Research conducted in the past has indicated that FoMO has a notable impact on the onset of psychopathological symptoms, which encompass emotional and behavioral disorders. Additionally, FoMO was found to be positively related to problematic buying behavior (Al-Saggaf, 2021; Aydin, et al., 2021). Therefore, it can be said that FoMO, which may be caused by fear of social exclusion, can compulsively influence consumers’ purchase decisions.

Furthermore, related studies show that people with FoMO and consumers who make compulsive purchases share many common characteristics, such as frequent presence on social media, the pursuit of innovations, and the search for rewarding experiences (Aydin et al., 2021; Salem, 2015). The similarities between these traits imply that there could be a connection between experiencing FoMO and exhibiting compulsive buying tendencies.

The current study suggests that an individual experiencing social exclusion constantly strives to eliminate this negative feeling and, as a result, engages in compulsive buying behavior by imitating the consumption style of others. From this point of view, the study predicts that FoMO may have a mediating role in the impact of social exclusion on compulsive buying behavior. Furthermore, no study was found that examined the mediating role of FoMO in the correlation between social exclusion and compulsive buying behavior. Based on a combination of empirical data and theoretical foundations, the following hypothesis is proposed.

- H1: Social exclusion is positively related to compulsive buying behavior, and this relationship is mediated by the FoMO.

### The moderating role of the narcissism

We argue that the correlation between social exclusion and FoMO may be influenced by the presence of narcissistic personality traits that act as moderators. Studies examining how narcissistic individuals respond to negative emotional states such as social exclusion have yielded different results. Some studies have shown that narcissistic individuals perceive social exclusion as a significant threat to their self-esteem, and that this perception may lead them to spend too much time on social media platforms and experience FoMO in its absence (Błachnio & Przepiórka, 2018; Chester & DeWall, 2016; Wu et al., 2013). Some studies have shown that narcissistic individuals are generally desensitized and ignore when they experience social exclusion and therefore feel less FoMO (Leung, 2013; Walters & Horton, 2015; Weiser, 2015). Other studies have also indicated that narcissistic individuals generally evaluate themselves in a superior position to other individuals and exhibit a tendency to always see themselves as more advanced (Hung, 2014; Kealy & Rasmussen, 2012). Therefore, compared to normal individuals, narcissistic individuals may have the belief that they are less likely to fall behind or lose touch if they experience social exclusion. The research hypothesis (H2) formulated in this framework will help us understand how narcissism can moderate the effects of social exclusion on FoMO.

Furthermore, our model suggests that narcissistic personality traits may moderate the potential link between FoMO and compulsive buying behavior. One of the salient characteristics of narcissistic individuals is that they tend to be self-centered, and their focus is largely on themselves, with diminished interest in other individuals (Hart et al., 2017). Therefore, it is reasonable to assume that narcissistic individuals who consider themselves superior in every way are less probable to be involved in compulsive buying behavior because they have less of a need to be accepted by others. Another research hypothesis (H3) based on these findings will help to show that narcissism plays a moderating role in the effect of FoMO on compulsive buying behavior.

On the other hand, social exclusion alone can explain only a small part of compulsive buying behavior (Harnish & Bridges, 2015). Thus, without knowledge of a person’s personality traits, it is difficult to understand how social exclusion influences compulsive buying behavior. Therefore, the inclusion of narcissism as a personality trait could provide deeper insight into the relationship between social exclusion and compulsive buying behavior. As mentioned earlier, studies of narcissistic individuals’ responses to negative emotional states such as social exclusion have so far yielded conflicting results (Harnish & Bridges, 2015; Lim, 2019; Ridgway et al., 2008; Rose, 2007). Others argue that narcissistic individuals ignore social exclusion, constantly view themselves as superior, and are therefore less likely to exhibit compulsive buying behavior because they do not tend to correct this situation when they experience social exclusion (Walters & Horton, 2015; Weiser, 2015). Others argue that narcissistic individuals ignore social exclusion, constantly view themselves as superior, and are therefore less likely to exhibit compulsive buying behavior because they do not tend to correct this situation when they experience social exclusion. Others argue that narcissistic individuals ignore social exclusion, constantly view themselves as superior, and are therefore less likely to exhibit compulsive buying behavior because they do not tend to correct this situation when they experience social exclusion. On this basis, we hypothesize the following:

- H2: Narcissism has a moderating effect on the relationship between social exclusion and FoMO.
- H3: Narcissism has a moderating effect on the relationship between FoMO and compulsive buying behavior.
- H4: Narcissism has a moderating effect on the relationship between social exclusion and compulsive buying behavior.

### Cultural differences of Denmark and Turkey

Cultural dimensions theory provides a framework for understanding how cultural values influence behavior and why individuals within a culture behave in a certain way (Hofstede, 2011). In this context, the individualism-collectivism and uncertainty avoidance dimensions of cultural dimensions theory were used to form the theoretical basis of this study. As per Hofstede’s theory of cultural dimensions, individualistic societies define the individual independently, while in collectivistic cultures, the individual obtains his identity through his family and group membership (Hofstede, 2011).

While collectivist societies prioritize the interests of the community and respond to the expectations of others, individualist societies prioritize personal aspirations and focus on individual success. In other words, in individualistic societies, the pronoun “I” represents the individual, whereas in collectivistic societies, the term “I” is used in conjunction with the cultural and social environment (Hung, 2014). On the individualism-collectivism dimension proposed by Hofstede, Denmark has a higher score than Turkey, suggesting that individual freedom is more prominent in Denmark, while family and community ties play a greater role in Turkey (Hofstede, 2011). Previous research has shown that egocentric and narcissistic personality traits are more prominent in individualistic societies such as Denmark (Akgün & Uysal, 2019; Hofstede, 2011). Foster et al. (2003) also found that individualism and narcissism are closely related, and narcissism is more prevalent in individualistic societies. Narcissistic societies such as Denmark tend to ignore negative emotional states more than other societies. Thus, when they encounter negative emotional states (social exclusion, FoMO, etc.), they are less likely to correct that emotional state and less probably to engage in problematic behaviors (compulsive buying behaviors, etc.) (Leung, 2013; Weiser, 2015). In this context, when examining cultural differences, it is assumed that the fact that Turkey and Denmark have different social characteristics within the individualism-collectivism dimension may differ in terms of whether there are regulatory effects in the direct and indirect relationships of narcissism within the proposed conceptual model.

On the other hand, negative situations cause more anxiety in societies with high levels of uncertainty avoidance. These societies try to get rid of anxiety, worry, and fear more quickly than societies with low levels of uncertainty avoidance. Therefore, they may exhibit problematic behaviors (Hofstede, 2011). In other words, societies experiencing high levels of uncertainty feel negative emotional states and exhibit another problematic behavior (such as compulsive buying behavior) to get rid of this feeling. In the uncertainty avoidance dimension suggested by Hofstede, Turkey has a higher value than Denmark, which means that Turkey feels more anxiety in the face of uncertain and negative situations and wants to get out of this situation faster (Hofstede, 2011). Therefore, in the face of negative situations (social exclusion, FoMO, etc.), Turkey may exhibit more problematic behavior (compulsive buying behavior, etc.) than Denmark. In this context, the effect of social exclusion and FoMO, which are negative emotional states, on compulsive buying behavior can be expected to be stronger in Turkey than in Denmark. On the other hand, FoMO is expected to play a mediating role in the impact of social exclusion on compulsive buying behavior in both countries, although differences in the strength of relationships are expected according to cultural values. The main reason for this is that, as suggested in Noelle-Neumann’s (1977) theory of the silence spiral, individuals often pursue the strategy of closely observing their environment and trying to understand the attitudes and behaviors prevalent in their environment in order to avoid the fear of social exclusion. This can lead to compulsive buying behavior by individuals who tend to imitate the behavior of others by being under constant social surveillance (Alabri, 2022; Noelle-Neumann, 1977). Therefore, FoMO can be expected to mediate the impact of social exclusion on compulsive buying behavior in both countries.

Finally, according to the We Are Social and Hootsuite report (2022), 70.8% of the population in Turkey, where uncertainty avoidance is higher, actively use social media, compared to 83.6% in Denmark. This ratio shows that more individuals use social media in Denmark. In their study, Buglass et al. (2017) found that in societies with a high rate of social media use compared to other societies, FoMO on developments is higher, and therefore, there is constant social follow-up. Therefore, it can be assumed that FoMO is higher in Denmark than in Turkey. On this basis, we hypothesize the following:

- H5: There is a statistical difference between social exclusion and country.
- H6: There is a statistical difference between compulsive buying behavior and country.
- H7: There is a statistical difference between FoMO and country.
- H8: There is a statistical difference between narcissism and country.

### The present study

**Fig. 1:**
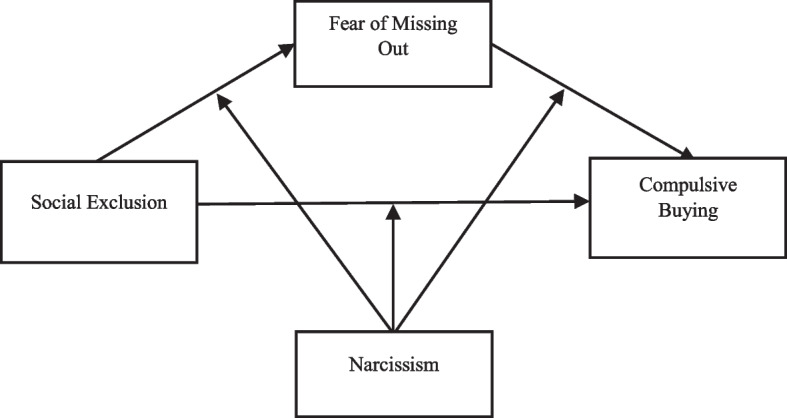
Conceptual model

Although the number of study papers addressing the correlations between social exclusion and compulsive buying behavior has increased in recent years, the relationships between mediators and regulatory variables between these constructs remain unexplored. From this perspective, the research aims to test a conceptual model and highlight the differences that may arise in the proposed conceptual model in two different cultures—Denmark and Turkey. This model explores the relationships between social exclusion, compulsive buying behavior, FoMO, and narcissism. Based on theoretical and empirical evidence, we hypothesize that social exclusion is a determinant of compulsive buying behavior, and that FoMO may mediate this relationship. Moreover, we anticipate the individuals with high levels of narcissism will exhibit stronger direct and indirect associations between social exclusion and compulsive buying behavior (Fig. 1).

## Method

### Participants

The study was conducted on a sample of university students who were currently residing in Turkey and Denmark. Participants willing to participate in the study between November 2022 and January 2023 were interviewed with a personal questionnaire. At the beginning of the study, the sample included 1020 university students, of which 520 participants were from Turkey and 520 from Denmark. However, 33 participants had to be eliminated from the analysis because of missing data (*n* = 29) and multiple responses (*n* = 4). The results regarding the demographic information of the participants are shown in Table 1.

**Table 1:** Demographic characteristics of participants

	**Denmark**		**Turkey**	
	Frequency	Percentage	Frequency	Percentage
**Gender**				
Male	266	53.1	250	49.4
Female	235	46.9	256	50.6
**Age**				
≤ 17	95	19.0	88	17.4
18–21	108	21.5	125	24.7
22–25	116	23.1	109	21.5
26–29	98	19.6	87	17.2
≥ 30	84	16.8	97	19.2

### Measures

#### Social exclusion

The study used the scale developed by Gilman et al. (2013) to measure the level of social exclusion of individuals. This scale consists of 19 items. In addition, the scale is a 5-point Likert scale (1 = strongly disagree to 5 = strongly agree). The scale contains statements such as “Usually other people do not care about me” and “Usually other people ignore my existence.” While the English version of the scale was used in Denmark, the version adapted into Turkish by Akın et al. (2016) was used in Turkey. The internal consistency value was found to be *α* = 0.893 for Denmark and *α* = 0.897 for Turkey.

#### Compulsive buying

To measure individuals’ compulsive buying tendencies, the scale developed by Andreassen et al. (2015) was used. This scale contains 7 items. In addition, the scale is a 5-point Likert scale (1 = disagree at all to 5 = agree completely). The scale contains the statements “I think people would be horrified if they found out about my shopping habits” and “I feel nervous and anxious on days when I don't go shopping.” While the English version of the scale was used in Denmark, the version adapted into Turkish by Bozdağ and Alkar (2018) was used in Turkey. The results showed that the value of internal consistency was *α* = 0.838 for Denmark and *α* = 0.742 for Turkey.

#### Fear of missing out (FoMO)

The scale developed by Przybylski et al. (2013) to measure FoMO level was used in this research. This scale consists of 10 items. In addition, the scale is a 5-point Likert scale (1 = strongly disagree to 5 = strongly agree). The scale includes the statements “When I am having a good time, it's important to me to share the details online” and “Sometimes I wonder if I spend too much time keeping track.” While the English version of the scale was used in Denmark, the version adapted into Turkish by Can and Satici (2019) was used in Turkey. The internal consistency value is *α* = 0.895 for Denmark and *α* = 0.885 for Turkey.

#### Narcissism

In the study, Gentile et al. (2013) used the NPI-13 (Narcissistic Personality Inventory13) scale to measure narcissism. The scale consists of 13 items, including statements such as “I have a strong will to power.” Participants rated the items on a 5-point scale from “strongly disagree” (1) to “strongly agree” (5). The survey conducted in Denmark used the original version of the scales (in English), whereas in Turkey, the version adapted to the Turkish language by Doğan and Çolak, 2020 was used, which has been shown to be valid and reliable. The results showed that the value of internal consistency was *α* = 0.887 for Denmark and *α* = 0.883 for Turkey.

### Procedures

The Ethics Committee of Atılım University approved the research to be conducted in accordance with ethical standards. Data collection was conducted in Denmark in November 2022 and in Turkey in January 2023. Only university students participated in the study. The study offered voluntary participation to the participants, with the assurance of keeping their information confidential. The participants who consented to take part in the study were given a self-administered questionnaire to fill out.

According to the EF English Proficiency Index (2022), Denmark ranks fifth in English proficiency among the 111 countries included in the study. While the original version of the scales (English) was used in Denmark, which has a very advanced level of English proficiency, the versions of the scales adapted to Turkish and tested for reliability were used in Turkey, which ranks 64th in the group of countries with low English proficiency in the study.

### Statistical analyses

Research data were collected face to face in both countries during a specific time period. The collected data were then manually entered into the SPSS package program. At this stage, erroneous data (missing responses (*n* = 29) and multiple responses (*n* = 4)) were identified and cleaned. Then, the reliability of the scales and the correlations between the descriptive statistics and the variables were examined. Data analysis was performed using PROCESS macro, which performs mediator and moderator effect analyses based on the SPSS program. To test these impact analyses, Hayes et al. (2017) recommend Model 4 and Model 59. More specifically, the study utilized Model 4 to examine the mediating function of FoMO. Subsequently, the study employed Model 59, a well-established model validated by numerous empirical studies, to investigate the moderating impact of narcissism on the direct and indirect associations between social exclusion and compulsive buying behavior (Frieder et al., 2018). To ensure the statistical significance and robustness of the effects, we utilized the bootstrapping method, which involved drawing 5000 resamples from the data and generating 95% bias-corrected confidence intervals for these effects (Hayes & Scharkow, 2013).

### Preliminary analyses

The variables’ means, standard deviations, and correlations are presented in Table 2. In addition, the results of the group comparison are also included in this table. The results of the correlation analysis for both countries show that social exclusion, FoMO, and narcissism are positively related to compulsive buying behavior. Furthermore, according to the available evidence, there seems to be a positive correlation between FoMO and social exclusion as well as narcissism.

**Table 2:** Pearson correlations and group comparison results of the main study variables (country)

Variables	1	2	3	4
**Turkey**				
1. SE	1			
2. CB	0.575**	1		
3. FoMO	0.576**	0.725**	1	
4. Narcissism	0.529**	0.506**	0.641**	1
Mean	3.16	3.29	3.34	3.31
Standard deviation	0.76	0.66	0.80	0.67
**Denmark**				
1. SE	1			
2. CB	0.512**	1		
3. FoMO	0.557**	0.619**	1	
4. Narcissism	0.592**	0.523**	0.532**	1
Mean	3.31	3.41	3.63	3.50
Standard deviation	0.76	0.71	0.83	0.77
**Group comparison (country)**				
Mean-Turkey	3.15	3.29	3.34	3.31
Mean-Denmark	3.30	3.40	3.63	3.49
t	3.14	2.62	5.66	4.09
p	0.002	0.009	< 0.001	< 0.001

*Note*: *N* = 1007, *SE* social exclusion, *CB* compulsive buying, *FoMO* fear of missing out. **p < 0.01, ***p < 0.001

An independent samples *t*-test was conducted to analyze whether the personality traits social exclusion, compulsive buying, FoMO, and narcissism showed a statistically significant difference as a function of the country variable. As a result of the analysis, a significant difference was found for the personality traits social exclusion (*p* < 0.01), compulsive buying (*p* < 0.01), FoMO (*p* < 0.001), and narcissism (*p* < 0.001) as a function of country (Table 2).

### Testing for mediation

As can be observed in Table 3, three different submodels were created in accordance with our model. Turkey and Denmark sample data were analyzed separately. Firstly, the first model analyzed how social exclusion affects compulsive buying behavior in the Turkish sample. This model links social exclusion to compulsive buying behavior, showing a positive relationship (*β* = 0.57, *p* < 0.001). Model 2 investigated whether social exclusion has an impact on the level of FoMO. The study demonstrates that social exclusion significantly predicts FoMO (*β* = 0.57, *p* < 0.001). Finally, Model 3 examines how social exclusion and FoMO collectively influence compulsive buying behavior. Social exclusion and FoMO positively impact compulsive buying behavior, according to the study’s findings (*β* = 0.23, *p* < 0.001); *β* = 0.59, *p* < 0.001), respectively). Thus, the study found significant correlations required for a mediation relationship in Turkey. Accordingly, the mediation effect of FoMO in the Turkey sample proved to be statistically significant (Table 4).

**Table 3:** Main effects on dependent variables

			**Turkey**			
	**Model 1 (CB)**		**Model 2 (FoMO)**		**Model 3 (CB)**	
	**β**	**t**	**β**	**t**	**β**	**t**
SE	0.57***	15.78	0.57***	15.84	0.23***	6.51
FoMO					0.59***	16.36
*R*^2^	0.33		0.33		0.56	
F	249.07		250.83		324.29	
			**Denmark**			
	***β***	***t***	***β***	***t***	***β***	***t***
SE	0.51***	13.30	0.55***	14.99	0.24***	5.90
FoMO					0.48***	11.81
*R*^2^	0.26		0.31		0.42	
F	176.95		224.72		182.82	

*Note*: *N* = 1007, *β* standardized estimate, *CB* compulsive buying, *SE* social exclusion, *FoMO* fear of missing out, ***p < 0.001

**Table 4:** Direct and indirect effects of social exclusion on compulsive buying

**Turkey**								
Total effects of social exclusion on compulsive buying					β	SE	LLCI	ULCI
					0.5751	0.0314	0.4335^a^	0.5568^a^
Direct effects of social exclusion on compulsive buying								
					0.2350	0.0311	0.1413^a^	0.2633^a^
Indirect effects of social exclusion on compulsive buying via FoMO								
Independent		Mediator		Dependent	β	SE	LLCI	ULCI
Social exclusion	** > **	FoMO	** > **	Compulsive buying	0.3401	0.0271	0.2410^a^	0.3480^a^
**Denmark**								
Total effects of social exclusion on compulsive buying					β	SE	LLCI	ULCI
					0.5116	0.0357	0.4043^a^	0.5444^a^
Direct effects of social exclusion on compulsive buying								
					0.2419	0.1496	0.1496^a^	0.2989^a^
Indirect effects of social exclusion on compulsive buying via FoMO								
Independent		Mediator		Dependent	β	SE	LLCI	ULCI
Social exclusion	** > **	FoMO	** > **	Compulsive buying	0.2698	0.0298	0.1930^a^	0.3092^a^

*Note*: *N* = 1007, *β* standardized estimate, *SE* standard error, *FoMO* fear of missing out. ^a^Total, direct, and indirect effects are significant if there is no 0 (zero) between LLCI and ULCI values (Hayes & Rockwood, 2020)

In the Danish sample analysis, Model 1 indicated a significant positive effect of social exclusion on compulsive buying behavior (*β* = 0.51, *p* < 0.001)). Additionally, Model 2 demonstrated a significant positive effect of social exclusion on FoMO (*β* = 0.55, *p* < 0.001)). Social exclusion (*β* = 0.24, *p* < 0.001)) and FoMO (*β* = 0.48, *p* < 0.001)) significantly predicted compulsive buying in Model 3. Therefore, the mediating effect of FoMO in Denmark is statistically significant as indicated (Table 4). Consequently, hypothesis 1 is supported for both countries.

As shown in Table 4, direct regression analyses between variables were performed to obtain the results of mediation analysis. The results of the analysis showed that FoMO plays a significant mediating role in the link between social exclusion and compulsive buying behavior in Turkey and Denmark (Turkey: *β* = 0.3401, *SE* = 0.0271, *p* < 0.001; Denmark: *β* = 0.2698, *SE* = 0.0298, *p* < 0.001). In this sense, FoMO mediates the positive effect of social exclusion on compulsive buying behavior in both countries.

### Testing for the moderated mediation

**Fig. 2:**
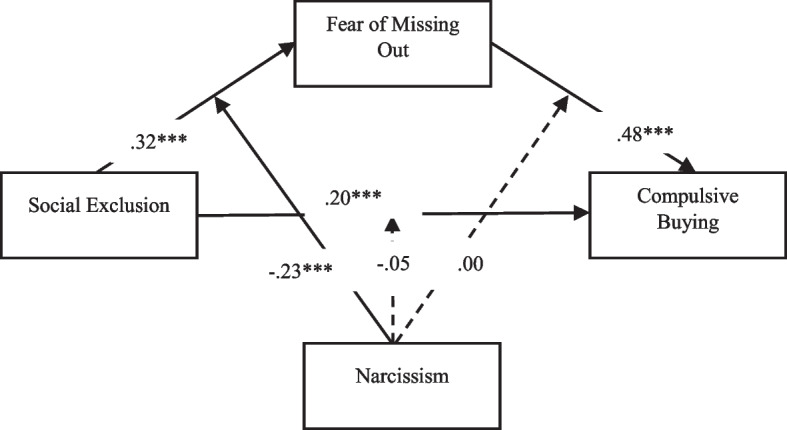
Results of moderated mediation (Turkey). ***p < 0.001

**Fig. 3:**
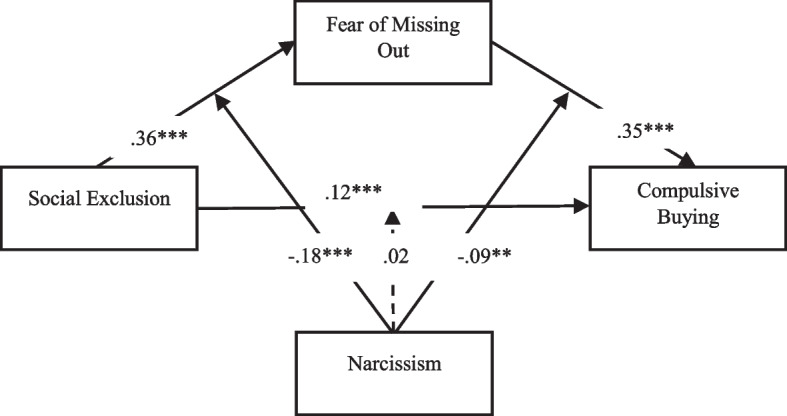
Results of moderated mediation (Denmark). **p < 0.01, ***p < 0.001

Model 59 was used to test hypotheses 2, 3, and 4. In the Turkish sample, the results showed that narcissism tended to moderate the link between social exclusion and FoMO (*B* =  − 0.23, *p* < 0.001) (Fig. 2). However, the effect of the interaction terms “social exclusion × narcissism” and “FoMO × narcissism” was not statistically significant (Table 5). In the Danish sample, the results also showed that narcissism tended to moderate the association between social exclusion and FoMO (*B* =  − 0.18, *p* < 0.001) (Fig. 3). In addition, narcissism (*B* =  − 0.09, *p* < 0.001) also appears to modulate the link between FoMO and compulsive buying behavior. However, the effect of the interaction term “social exclusion × narcissism” was not statistically significant (Table 5).

**Table 5:** Testing the moderated mediation effects of social exclusion on compulsive buying

	Turkey				Denmark			
	B	SE	LLCI	ULCI	B	SE	LLCI	ULCI
FoMO								
SE	0.32	0.04	0.25^a^	0.40^a^	0.36	0.05	0.26^a^	0.45^a^
Narcissism	0.51	0.05	0.42^a^	0.60^a^	0.30	0.05	0.21^a^	0.40^a^
SE × narcissism	− 0.23	0.05	− 0.32^a^	− 0.13^a^	− 0.18	0.05	− 0.28^a^	− 0.07^a^
*R*^2^	0.51				0.39			
F	174.83				104.78			
CB								
SE	0.20	0.03	0.14^a^	0.27^a^	0.12	0.04	0.04^a^	0.21^a^
FoMO	0.48	0.03	0.41^a^	0.54^a^	0.35	0.04	0.28^a^	0.42^a^
Narcissism	0.00	0.03	− 0.08^a^	− 0.08^a^	0.19	0.04	0.11^a^	0.27^a^
SE × narcissism	− 0.05	0.05	− 0.15	0.05	0.02	0.05	− 0.08	0.12
FoMO × narcissism	0.00	0.05	− 0.09	0.10	− 0.09	0.04	− 0.18^a^	− 0.00^a^
*R*^2^	0.56				0.45			
F	129.67				82.31			

*Note*: *N* = 1007, *FoMO* fear of missing out, *SE* social exclusion, *CB* compulsive buying. ^a^Total, direct, and indirect effects are significant if there is no 0 (zero) between LLCI and ULCI values (Hayes & Rockwood, 2020)

On the other hand, in addition to these analyses, we used the Johnson-Neyman approach to see at which levels of narcissism the impact of social exclusion on compulsive buying behavior differs. Table 6 shows that although the conditional indirect effect is significant in all cases, it varies in magnitude depending on the degree of narcissism. The effect is lowest for individuals with high narcissism, higher for individuals with medium narcissism, and highest for individuals with low narcissism.

**Table 6:** Conditional indirect effect of social exclusion on compulsive buying via FoMO at different levels of narcissism

Level of narcissism	Turkey				Denmark			
	Indirect effect	Boot SE	LLCI	ULCI	Indirect effect	Boot SE	LLCI	ULCI
**Low** (1 SD below mean)	0.2242	0.0325	0.1612^a^	0.2898^a^	0.2058	0.0382	0.1350^a^	0.2856^a^
**Medium** (Mean)	0.1536	0.0246	0.1065^a^	0.2027^a^	0.1249	0.0231	0.0828^a^	0.1737^a^
**High** (1 SD above mean)	0.0821	0.0306	0.0231^a^	0.1438^a^	0.0625	0.0248	0.0206^a^	0.1180^a^

*Note*: *N* = 1007. ^a^Total, direct, and indirect effects are significant if there is no 0 (zero) between LLCI and ULCI values (Hayes & Rockwood, 2020)

## Discussion

Based on Hofstede’s theory of cultural dimensions, social media use is lower in Denmark, where uncertainty avoidance is lower, than in Turkey (Hofstede, 2011; We Are Social & Hootsuite, 2022). Buglass et al. (2017) found in their study that societies with a high rate of social media use are more afraid of missing out on developments compared to other societies, and therefore, they are constantly in social follow-up. Similarly, societies with a high rate of social media use are expected to exhibit more compulsive buying behavior compared to other societies (Billieux, 2012; Krajewska-Kułak et al., 2012). The main reason for this could be that they feel more deficits when exposed to more content (Billieux, 2012). Therefore, FoMO and compulsive buying behavior can be expected to be higher in Denmark than in Turkey. On the other hand, Denmark has a higher value than Turkey in the individualism-collectivism dimension suggested by Hofstede, which indicates that individual freedom is more prominent in Denmark, while family and community ties are more prominent in Turkey (Hofstede, 2011). Previous research has shown that narcissistic personality traits are more prominent in individualistic societies such as Denmark (Akgün & Uysal, 2019; Foster et al., 2003; Hofstede, 2011). Therefore, the results of the study are in line with the findings in the literature. Finally, it can be said that narcissistic individuals are more prone to social exclusion (Bushman & Baumeister, 1998; Spector, 2011). The reason for this can be seen as the fact that narcissistic individuals generally consider themselves superior to other individuals, always see themselves as more advanced, and underestimate other individuals (Lamarche & Seery, 2019; Miller & Campbell, 2008; Thomaes & Brummelman, 2016). In this context, it is an expected result that social exclusion is higher in Denmark, which has a narcissistic society characteristic.

**Fig. 4:**
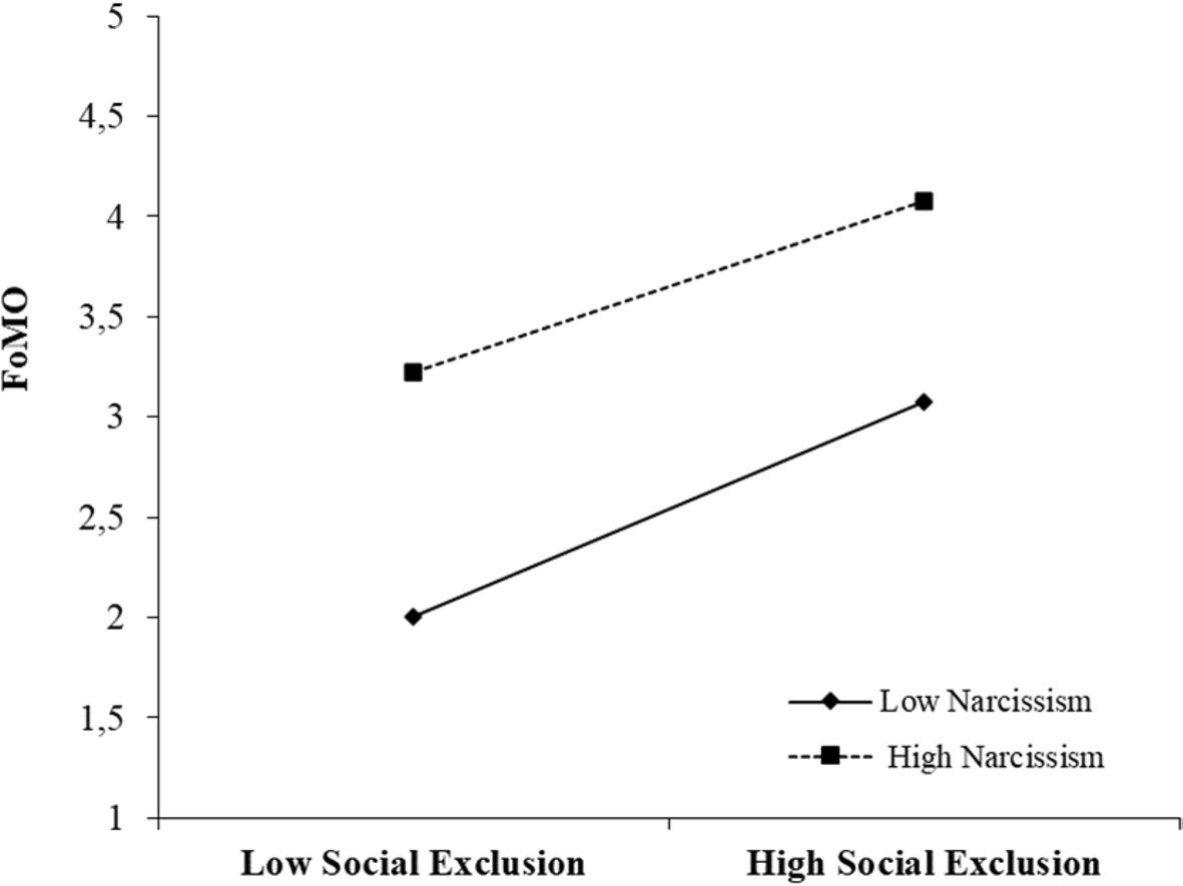
The moderating role of narcissism in the relation between social exclusion and FoMO (Turkey)

**Fig. 5:**
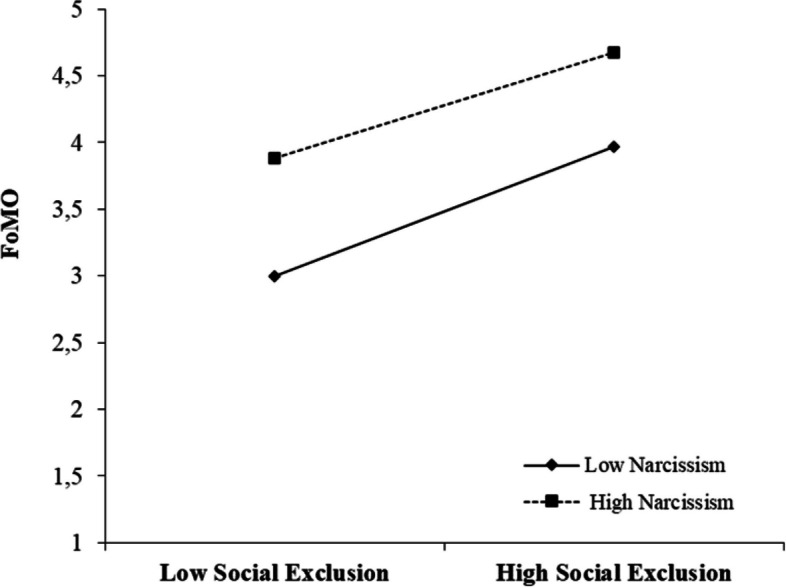
The moderating role of narcissism in the relation between social exclusion and FoMO (Denmark)

**Fig. 6:**
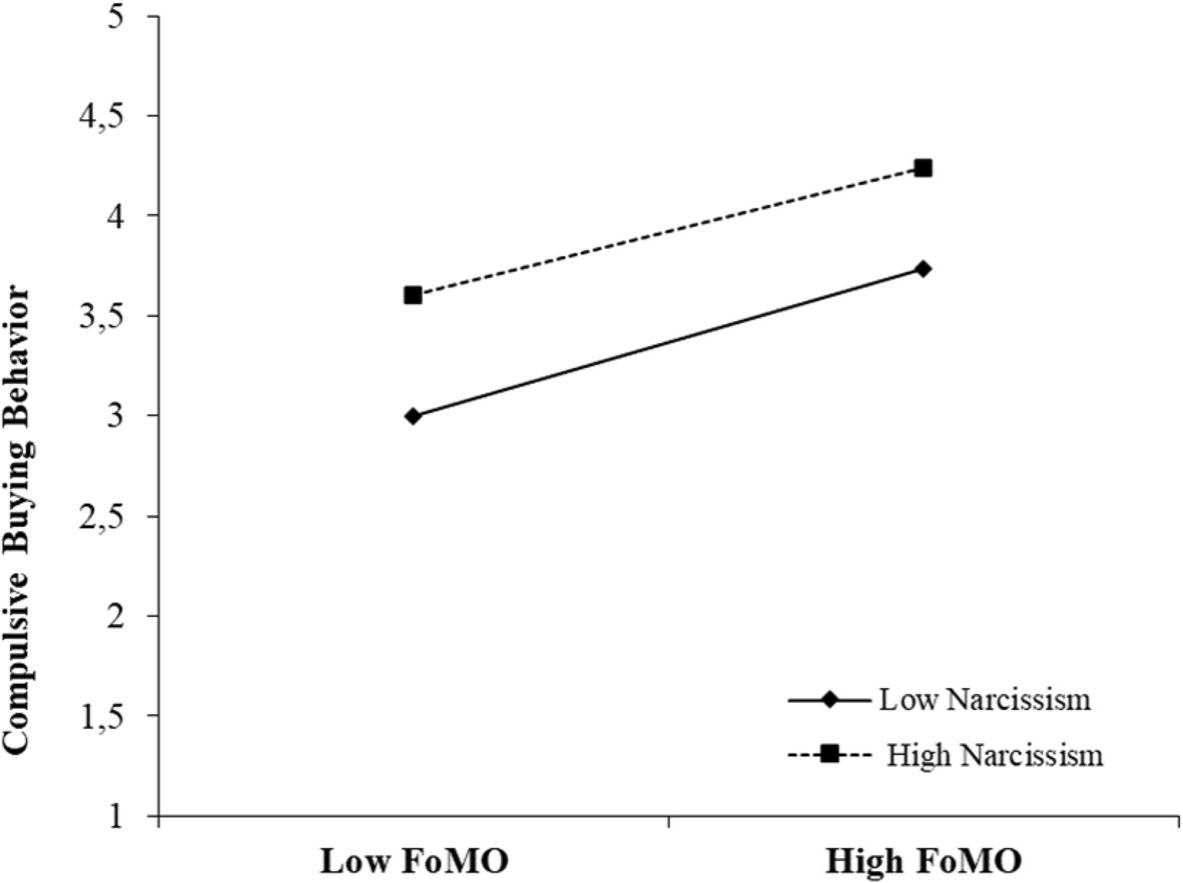
The moderating role of narcissism in the relation between FoMO and compulsive buying behavior (Denmark)

On the other hand, earlier research has indicated that compulsive buying behavior can be influenced by social exclusion (Han, 2020; Harnish & Bridges, 2015; Leary, 1990). However, prior studies have not thoroughly investigated the mediating and regulating mechanisms that explain how social exclusion impacts compulsive buying behavior. A moderated mediation model was applied in the study to investigate possible relationships between variables. In both country samples, FoMO played a mediating role between social exclusion and compulsive buying behavior (Table 7). In addition, narcissism had a moderate influence on the correlation between social exclusion and FoMO in both countries (Figs. 4 and 5 ). Unlike in the Turkish sample, narcissism also moderated the effect between FoMO and compulsive buying behavior in the Danish sample (Fig. 6). Therefore, the findings of this research expand our understanding of how social exclusion is related to compulsive buying behavior and help us uncover any country differences that may arise. On the other hand, the study provides a broad perspective for developing preventive measures to reduce problematic behaviors that may arise as a result of the negative effect of social media on individuals worldwide.

**Table 7:** Summary of hypothesis testing

Hypothesized relationship	Mediation	Moderation	Results (Turkey)	Results (Denmark)
**H1**: SE → CB and SE → FoMO → CB	FoMO	-	Accepted	Accepted
**H2:** SE → FoMO	-	Narcissism	Accepted	Accepted
**H3:** FoMO → CB	-	Narcissism	Rejected	Accepted
**H4:** SE → CB	-	Narcissism	Rejected	Rejected
**H5:** SE (country)	-	-	Accepted	
**H6:** CB (country)	-	-	Accepted	
**H7:** FoMO (country)	-	-	Accepted	
**H8:** Narcissism (country)	-	-	Accepted	

*Note*: *SE* social exclusion, *CB* compulsive buying, *FoMO* fear of missing out

### The mediating role of FoMO

As expected, this research supports that FoMO acts as a mediator between social exclusion and compulsive buying behavior in both countries. That is, social exclusion influences compulsive buying behavior via FoMO. Examining the potential mediating effect of FoMO, a negative emotional state, in the correlation between social exclusion and compulsive buying behavior, may provide insight into the cognitive mechanisms underlying compulsive buying behavior. These cognitive mechanisms could contribute to the development of techniques to avoid compulsive buying behavior.

Previous studies have shown that negative moods (FoMO, stress, anxiety, etc.) can lead people to engage in compulsive buying behavior (Aydin et al., 2021; Saranya, 2019). As suggested in Noelle-Neumann’s (1977) theory of the silence spiral, individuals who fear being ostracized turn to popular views and styles in their environment and constantly try to understand which styles are popular. This theory also assumes that individuals may experience FoMO even if they are not in that environment. On the other hand, it has been found that these individuals tend to imitate others in order to express themselves in a socially appropriate manner and be accepted in that group and therefore may engage in problematic buying behavior (compulsive buying, etc.) (Noelle-Neumann, 1977). Indeed, the study shows that social exclusion has a positive effect on FoMO in both countries, which in turn may increase compulsive buying behavior. Consequently, it can be said that FoMO is an important mechanism linking social exclusion and compulsive buying behavior.

### The moderating role of narcissism

The results of the study show that narcissism has a moderate influence on the correlation between social exclusion and FoMO in both countries. While this result is consistent with our predictions, it suggests that narcissism attenuates rather than strengthens the relationship between social exclusion and FoMO. However, considering that an alternative explanation for these results was also considered, we can say that the results are generally consistent with the existing literature. Alabri (2022) emphasized that in situations where individuals feel socially excluded, FoMO on developments increases. For this reason, they tend to follow social followers constantly and often use social media platforms to satisfy this need. Bergman et al. (2011) found that narcissistic individuals spend more time on social media platforms but do so with the goal of promoting their own content and making status updates rather than following others, as is the case with other individuals. Kealy and Rasmussen (2012) explain this situation by saying that narcissistic individuals prioritize content for themselves when using social media, their interest in others decreases, and they become desensitized and display a completely egocentric attitude. When narcissistic individuals experience social exclusion, they ignore it. They always think they are better. So, if they experience social exclusion, they may not participate in FoMO’s social monitoring of developments like normal individuals (Leung, 2013; Walters & Horton, 2015; Weiser, 2015). Overall, these results provide an important contribution to our understanding of how narcissism moderates the effects of social exclusion on FoMO. On the other hand, contrary to expectations, narcissism does not play a moderating role in the effect of social exclusion on compulsive buying behavior in either country. The significant impact of social exclusion on compulsive buying behavior could explain this result (Han, 2020). However, further research is needed to draw firm conclusions about the extent to which narcissism influences the relationship between social exclusion and compulsive buying behavior.

In addition, another remarkable result obtained from the study is that narcissism does not moderate the relationship between FoMO and compulsive buying behavior in Turkey; however, it weakens this relationship in Denmark. In the context of Hofstede’s cultural dimensions, Turkey is characterized as a collective society, while Denmark is characterized as an individual society (Akgün and Uysal, 2019; Hofstede, 2011). Individual societies exhibit more narcissistic personality traits than collective societies (Hofstede, 2011). Our results show that individuals living in Denmark, an individualistic society, exhibit more narcissistic personality traits than individuals living in Turkey, a collective society (*p* < 0.001). Narcissistic individuals often tend to evaluate themselves as superior to the people around them and position their own abilities at a higher level than others (Hart et al., 2017). Individuals with this trait often firmly embrace the idea of believing that they are always one step ahead of others (Hung, 2014; Kealy & Rasmussen, 2012). Compared to normal people, narcissistic people therefore tend to engage in less compulsive buying behavior because they believe they are less likely to be left behind or to miss out on developments. On the other hand, narcissistic individuals are often unaware of who they really are and how they are perceived by others. They tend to view their own needs and goals as superior and paramount, often above the emotional or physical needs of others or societal norms (Kealy & Rasmussen, 2012). These individuals view the expectations or opinions of those around them as secondary or unimportant and are determined to focus on their own goals (Twenge & Campbell, 2003). Therefore, narcissistic individuals have less fear of social evaluation compared to normal individuals (Hart et al., 2017). Considering the fact that narcissistic individuals, who believe they are superior in everything, have less need to be accepted by other people, it can be assumed that they are less prone to compulsive buying behavior. Therefore, these results help us understand how narcissism moderates the impact of FoMO on compulsive buying behavior in Denmark, which has more narcissistic traits than Turkey (Table 2).

### Limitations and practical implications

As is inherent in any study, this particular research has some limitations that warrant consideration. The first is that the data we used were self-reported, which may obscure the true responses. In future studies, more objective measurements, such as experimental studies, should be conducted to validate the model proposed in the current research. Second, the study involves students from a university as its sample. To better understand how social exclusion is related to compulsive buying behavior, future studies could include participants from all age groups. Third, this study only examined FoMO as a mediator, but as mentioned in previous sections, the effect of social exclusion on compulsive buying behavior may also be mediated by negative mood states (e.g., social anxiety, stress). Finally, due to cost and time constraints, the study was conducted only among university students. Future studies could include all age groups to reveal age differences in relation to the proposed model.

On the other hand, the study has important practical and theoretical contributions. The main contribution of the research is that it reveals the possible correlation between social exclusion and compulsive buying behavior and develops a mechanism that can contribute to the literature. In addition, the study is important as it reveals the country-specific differences that may occur on the proposed conceptual model since it was conducted in Turkey and Denmark. In addition, the study emphasizes the need for educational programs that help to get rid of the feeling of social exclusion that leads to negative behaviors. These proposed programs should focus on how individuals can cope with their negative emotional states. In addition, the study emphasizes that the personality trait of narcissism has an important place in the behavior of the individual and to what extent it can change the negative emotions experienced by the individual after social exclusion. This situation emphasizes the importance of an accurate self-perception of the individual. In this context, therapists should be informed about social exclusion, FoMO, compulsive buying behavior, and narcissistic personality traits that create negative emotional states on the individual.

## Conclusions

This study’s exploration of a moderated mediation model enhances our understanding of the correlation between social exclusion and compulsive buying behavior by providing a novel perspective and valuable insights into the mechanisms and conditions that shape this association. The study found that in both country samples, the mediator between social exclusion and compulsive buying behavior is FoMO. Furthermore, the results suggest that narcissism plays a moderating role in indirect effects. The conclusion of the study shows that narcissism has a moderating effect on the correlation between social exclusion and FoMO in both countries. Moreover, another noteworthy result obtained from the study is that narcissism does not moderate the correlation between FoMO and compulsive buying in Turkey; however, it weakens this relationship in Denmark. It expands our knowledge of the connection between social exclusion and compulsive buying behavior by identifying the moderating role of narcissism in this study.

## Data Availability

The datasets generated during and/or analyzed during the current study are available from the corresponding author on reasonable request.

## Abbreviations

SE: Social exclusion
CB: Compulsive buying
FoMO: Fear of missing out
